# The Epstein-Barr virus latent membrane protein-1 (LMP1) 30-bp deletion and XhoI-polymorphism in nasopharyngeal carcinoma: a meta-analysis of observational studies

**DOI:** 10.1186/s13643-015-0037-z

**Published:** 2015-04-13

**Authors:** Vivaldo G da Costa, Ariany C Marques-Silva, Marcos L Moreli

**Affiliations:** Post-Graduation Program in Applied Health Sciences, Federal University of Goiás, BR 364, Km 192, Industrial Park, Jataí, Brazil; Virology Laboratory, Federal University of Goiás, BR 364, Km 192, Industrial Park, Jataí, Brazil

**Keywords:** Epstein-Barr virus, Nasopharyngeal carcinoma, Meta-analysis, Systematic review, Latent membrane protein-1, XhoI-loss

## Abstract

**Background:**

Epstein-Barr virus (EBV) is considered to be closely associated with nasopharyngeal carcinoma (NPC), in which EBV-encoded latent membrane protein 1 (LMP1) was found to have an oncogenic role. However, the results published on the LMP1 polymorphism are inconsistent. In the present study, we performed a meta-analysis to determine the frequency of the associations and a more precise association between NPC and EBV LMP1 gene variants (30-bp deletion (del)/XhoI-loss).

**Methods:**

Eligible articles met the inclusion/exclusion criteria and were identified in the following electronic databases: PubMed, ScienceDirect, and SciELO. Consequently, the data of interest were extracted and plotted in a table to calculate the frequency and odds ratio (OR) of the outcomes of interest (30-bp del-LMP1/XhoI-loss) in patients with NPC. Study quality (Newcastle-Ottawa Scale (NOS)), publication bias, and heterogeneity were assessed.

**Results:**

Thirty-one observational studies were included with a total of 2,846 individuals (NPC, *n* = 1,855; control, *n* = 991). The risk of bias in relation to study quality evaluated by NOS was considered low. The pooled estimate of the frequency of 30-bp del-LMP1 and XhoI-loss in patients with NPC was 77% (95% confidence interval (CI): 72 to 82) and 82% (95% CI: 71 to 92), respectively. There was an association between 30-bp del-LMP1 and NPC susceptibility (OR = 2.86, 95% CI: 1.35 to 6.07, *P* = 0.00). Similarly, there was an association between XhoI-loss and NPC (OR = 8.5, 95% CI: 1.7 to 41, *P* = 0.00). However, when we analyze the co-existence of the 30-bp del-LMP1 and XhoI-loss in patients with NPC, there was no association (OR = 1.09, 95% CI: 0.06 to 18.79, *P* = 0.002).

**Conclusions:**

Our results suggest an association between the 30-bp del-LMP1 and XhoI-loss with NPC susceptibility. However, our data should be interpreted with caution because the sample size was small, and there was heterogeneity between the studies. Thus, future studies are needed with adjusted estimates to simultaneously evaluate multiple factors involved in the development of NPC.

**Systematic review registration:**

PROSPERO CRD42014013496.

**Electronic supplementary material:**

The online version of this article (doi:10.1186/s13643-015-0037-z) contains supplementary material, which is available to authorized users.

## Background

Nasopharyngeal carcinoma (NPC) is an aggressive tumor associated with Epstein-Barr virus (EBV) that initially affects the layer of epithelial cells in the nasopharynx and preferably arises in the Rosenmüller’s fossa [1,2]. NPC is also defined as a peculiar type of head and neck cancer due to its clinical status, etiology, pathology, epidemiology, and manner of response to treatment [3,4]. In view of these factors, EBV and NPC have very distinct epidemiological and clinical patterns, given that EBV is ubiquitous and causes a latent infection in 95% of the world population, with the majority of these infections being benign [5]. However, the worldwide incidence of NPC is considered rare, and affected individuals for many years had a poor prognosis that reflected in a reduced survival rate, but actually the survival of NPC patients is relatively high under the current treatment protocol and the introduction of intensity-modulated radiotherapy (IMRT) [6].

Although NPC endemicity is rare in most parts of the world [7], there is a striking geographic and ethnic distribution of cases characterized by existing areas of high endemicity (Southeast Asia), intermediate endemicity (Eastern Mediterranean and North Africa), and low endemicity (Americas). According to data provided by GLOBOCAN 2012 [8], in the same period in the world, the total number of NPC was 86,691 cases (age-standardized rate (ASR), 1.2/100,000)), while the number of deaths was 50,831 (ASR = 0.7/100,000). Among these cases, China was the country that contributed the most to these indices because it had the highest total number (33,198, ASR = 1.9/100,000) and deaths (20,404, ASR = 1.2/100,000) by NPC, followed by Indonesia (13,084 cases, ASR = 5.6/100,000 and 7,391 deaths with ASR = 3.3/100,000) and Vietnam (4,931 cases, ASR = 5.4/100,000 and 2,885 deaths with ASR = 3.3/100,000).

EBV infects the mucosal epithelium and B cells and consequently establishes a latent infection, and aberrant infection by EBV can to cause the appearance of tumors in these locations of replication [9]. Hence, infected cells may present physiological changes that result from the expression of latent genes, including mainly EB-encoded early RNAs (EBER), EBV nuclear antigens (EBNA1/2/3a,b,c) and latent membrane protein 1 or 2 (LMP1/2) [10].

The first protein from EBV to have its oncogenic properties empirically demonstrated was LMP1 [11], which is expressed on the cell surface, where it spontaneously aggregates to form a constitutively activated receptor, acting as a member of the tumor necrosis factor receptor (TNF-R) family and allowing LMP1 to exert an influence on the cell through interactions with different cellular molecules involved in an intracellular signaling cascade [12-15]. Consequently, a number of studies have shown the involvement of LMP1 in the pathogenesis of NPC [16,17], which involves the following factors: 1) an inhibition of apoptosis in the infected cells by the upregulation of Bcl-2 and A-20 genes [13,15,18]; 2) a modulation of the morphology and motility of epithelial cells [19]; 3) a downregulation of several suppressors for metastases [20]; 4) a promotion of angiogenesis [21]; and 5) an induction of the expression of proinflammatory cytokines, among other mechanisms [22,23]. LMP1 is an integral membrane protein that can be divided into three domains: 1) a short cytoplasmic N-terminal tail (amino acids 1 to 23); 2) six transmembrane alpha-helical of hydrophobic nature (amino acids 24 to 186); and 3) a long cytoplasmic C-terminal tail (amino acids 187 to 386), with this region having the highest LMP1 signaling activity. Therefore, the long cytoplasmic C-terminal tail has three distinct functional domains: C-terminal activation regions 1, 2, and 3 (CTAR1/2/3) [24,25].

The LMP1 gene has been shown to have polymorphisms, among which the occurrence of a 30-bp deletion is prominent, as compared with the prototype B95-8 LMP1, and occurs near the end of the cytoplasmic C-terminal tail and close to the functional domain CTAR2. This feature deletion has been studied, and it has been established that the 30-bp deletion results in an increased oncogenic activity of infected cells and results in a more aggressive phenotype of EBV-associated tumors [26,27]. In addition, another mutation observed in the LMP1 gene, albeit a point mutation, occurs at nucleotide position G169425T, resulting in the loss of a restriction site known as XhoI, which is present in the cytoplasmic N-terminal tail. Thus, the XhoI polymorphism has been commonly found in samples from patients with NPC but is absent in samples from healthy individuals [28,29]. However, in general, the results of the studies are ambiguous regarding the association of these variants in the EBV LMP1 gene with the risk of developing NPC. In this sense, this meta-analysis was performed to solve the problem of inadequate statistical power and of the controversial and ambiguous results.

## Methods

We followed the Preferred Reporting Items for Systematic Reviews and Meta-Analyses (PRISMA) protocol, which provides rules and guidelines for systematic reviews and meta-analysis [30]. The PRISMA checklist is shown in Additional file 1. In addition, we prospectively recorded the study protocol in PROSPERO with the registration number CRD42014013496, available on the website http://www.crd.york.ac.uk/PROSPERO/display_record.asp?ID=CRD42014013496#.VBg8HoctBdg.

### The identification and eligibility of relevant studies

To address our study hypothesis, the following electronic databases were searched: PubMed, ScienceDirect, and SciELO. The selection period lasted until the beginning of September 2014, and the language of the studies to be selected should be expressed in English, Spanish, or Portuguese. Therefore, in these three electronic databases, the following keywords related to our research topic were added: Epstein-Barr virus OR EBV OR virus AND cancer OR tumor OR neoplasia OR nasopharyngeal carcinoma AND LMP1 30-bp deletion OR LMP1 variant OR BNLF1 AND XhoI polymorphism OR XhoI variants. We also adopted another strategy to search the data to reduce possible selection bias, which was the recovery of all references of ‘pre-selected’ studies to retrieve articles of interest available only in other electronic databases. Finally, the selection criteria were as follows: 1) cohort or case–control studies that analyzed the 30-bp deletion and/or XhoI-loss present in the EBV LMP1 gene of patients with NPC; and 2) studies that showed the variables of interest as previously stated were selected regardless of the place of origin of the search, age, and sex as well as the histological type of NPC presented by these individuals. The exclusion criteria included the following: 1) review studies; 2) analysis only of other cancers ‘non-NPC’; 3) duplicated previous publications; and 4) studies that did not disclose the variables of interest as the frequency of the 30-bp deletion or the XhoI polymorphism in the EBV LMP1 gene separately for patients with NPC.

### Data extraction and quality assessment

The researchers independently reviewed and extracted information from the eligible studies according to the inclusion and exclusion criteria previously highlighted. Thus, the following data were extracted: the identification and design of the study, the year of publication, the number of subjects and controls who had NPC, the types of controls (healthy or pathological), the study site, the age and sex of the research subjects, the frequency of the outcomes of interest as the 30-bp deletion or XhoI-loss from the EBV LMP1 gene. To assess the methodological quality of observational studies was used Newcastle-Ottawa Scale (NOS) in which a study was judged on three categories: selection (four items, one star each), comparability (one item, up to two stars), and exposure/outcome (three items, one star each). A ‘star’ presents a ‘high-quality’ choice of individual study. The full score was 10 stars, and the high-quality study was defined as a study with ≥6 awarded stars [31].

### Statistical analysis

The dichotomous data of the case–control studies were extracted and plotted in a 2 × 2 table to give the individual and combined odds ratios (OR). For the cohort studies, which have binary data, the variables were extracted to calculate an estimate of the frequency of the outcomes of interest, and a confidence interval (CI) of 95% was used whenever possible. The *I*^2^ index was used to evaluate the existence of heterogeneity between the studies; for cases where there were significant differences in terms of heterogeneity (*I*^2^ = 75% to 100%, *P* < 0.05) [32], the random-effect model was used for the individual and combined analysis of the data. We conducted a sensitivity analysis to test the effect of the individual influence of each study on the overall estimate, and a subgroup analysis was also performed to reduce the existence of heterogeneity. Furthermore, we evaluated the existence of publication bias by Begg’s funnel plot [33] and by Egger’s test [34]. The funnel plot for the case–control studies was developed from the standard error of the log (OR) for each analysis against the log (OR), whereas the funnel plot of the cohort studies was plotted from the standard error log (percent) against the log (percent). For all of the procedures of the meta-analysis, STATA IC/64 version 13.1 software (Stata Corporation, College Station, Texas, USA) was used.

## Results

Initially, during our search for articles in the electronic databases, 1,280 reference studies were found (see Additional file 2). Subsequently, due to the selection of the inclusion and exclusion criteria, there was a refinement to 75 reference studies (see Additional file 3). Finally, 31 eligible studies remained, which constituted our database for conducting the present meta-analysis (Table 1) [29,35-64]. A total of 2,846 subjects were included in these studies, among which 1,855 and 991 individuals were placed in NPC and control groups, respectively. Regarding the sex of the individuals, there was a male predominance with a male/female ratio of 2.73 [39-42,46,49,53,54,57]. The age of the participants ranged from 8 to 87 years; however, the predominant age was approximately 50 years old [35,40,41,43,46,49,53-55]. Most of the participants came from Asia (approximately 71%) [29,35,36,38-41,43,46,47,49-51,53,55,57-59,61-64], followed by Europe (26%) [29,36,37,48,54,56,62,63], North Africa (19%) [42,44,52,56,60,62], and America (13%) [45,54,62,63]. Similarly, the samples were from at least 12 countries, consisting mainly of China, Taiwan, Russia, Malaysia, Tunisia, Indonesia, Thailand, Serbia, and Morocco. With regard to case–control studies, all had an NOS score of 7. In the cohort studies, 5 (42%) were of high quality (NOS score ≥6), with an average NOS score of 5 (see Additional file 4). Thus, the risk of bias in relation to study quality evaluated by NOS was considered low with a score ranging from 4 to 7.

**Table 1:** **The characteristics of the studies included in the meta-analysis**

**Trial (year)**	**Location**	**Design**	**NPC 30-bp del-LMP1 EBV**			**XhoI-loss LMP1 EBV**			**Control group**		
			**Present**	**Not present**	**Type of sample**	**Present**	**Not present**	**Type of sample**	***N***	**Type of sample**	**Type of control**
Senyuta et al*.* 2014 [35]	Russia	Case–control	4	18	Biopsy				15	PBMC	HD
Gurtsevitch et al*.* 2013 [36]	Russia	Case–control	3	18	Biopsy				13	PBMC	Non-NPC
Banko et al*.* 2012 [37]	Serbia	Case–control	1	15	Biopsy				30	PBMC	IM
Li et al*.* 2009 [38]	China	Case–control	43	7	Biopsy				56	PBMC	HD
See et al*.* 2008 [39]	Malaysia	Case–control	19	15	Biopsy	34	5	Biopsy	8	Biopsy	Non-NPC
Tang et al*.* 2008 [40]	China	Cohort	18	3	Biopsy	21	0	Biopsy			
Tiwawech et al*.* 2008 [41]	Thailand	Case–control	44	31	PBL				44	PBMC	Non-NPC/HD
Ayadi et al*.* 2007 [42]	Tunisia	Cohort	41	6	Biopsy	0	47	Biopsy			
Chang et al*.* 2006 [43]	Taiwan	Cohort	446	88	Biopsy						
Dardari et al*.* 2006 [44]	Morocco	Case–control	51	10	Biopsy				17	PBMC	HD
Chabay et al*.* 2004 [45]	Argentina	Case–control	3	1	Biopsy				15	Biopsy/PBMC	Non-NPC
Min et al*.* 2004 [46]	China	Case–control	87	12	Biopsy				46	PBMC	HD
Nurhantari et al*.* 2003 [47]	Indonesia	Cohort	22	33	Biopsy						
Plaza et al*.* 2003 [48]	Spain	Case–control	18	9	Biopsy				40	-	EBV-RC
Tan et al*.* 2003 [49]	Malaysia	Case–control	25	0	Biopsy	25	2	Biopsy	12	Biopsy	
Zhang et al*.* 2002 [50]	China	Case–control	36	7	Biopsy				36	TW	HD
Hahn et al*.* 2001 [51]	Russia	Case–control	0	7	Biopsy				10	PBMC	HD
Henry et al*.* 2001 [52]	Tunisia	Cohort	3	3	Biopsy	0	6	Biopsy			
Kuo et al*.* 2001 [53]	Taiwan	Cohort	2	0	Biopsy						
D’Addario et al*.* 2000 [54]	Canada	Cohort	9	14	Biopsy						
Cheung et al*.* 1998 [55]	China	Cohort	34	3	Biopsy						
Grunewald et al*.* 1998 [56]	Multicenter	Case–control	46	18	Biopsy				45	PBMC	HD
Sung et al*.* 1998 [57]	China	Cohort	30	12	Biopsy	22	6	Biopsy			
Cheung et al*.* 1996 [58]	China	Case–control	69	5	Biopsy	5	0	Biopsy	11	Biopsy	Non-NPC
Khanim et al*.* 1996 [29]	Multicenter	Case–control	24	4	Biopsy	19	9	Biopsy	14	Biopsy/PBMC	Non-NPC
Chang et al*.* 1995 [59]	Taiwan	Case–control				48	0	Biopsy	25	Biopsy/TW	HD
Bouzid et al*.* 1994 [60]	North Africa	Cohort				1	12	Biopsy			
Jeng et al*.* 1994 [61]	Taiwan	Case–control				25	7	Biopsy	79		Non-NPC
Miller et al*.* 1994 [62]	Multicenter	Cohort	9	4	Biopsy						
Abdel-Hamid et al*.* 1992 [63]	Multicenter	Case–control				33	18	Biopsy	19	-	Non-NPC
Hu et al*.* 1991 [64]	Multicenter	Cohort				38	18	Biopsy			

*Abbreviations:* PBMC = peripheral blood mononuclear cell; PBL = peripheral blood lymphocyte; HD = healthy donors; IM = infective mononucleosis; TW = throat washing samples; NPC = nasopharyngeal carcinoma.

### A meta-analysis to estimate the frequency of the 30-bp deletion and XhoI-loss (LMP1 EBV) in samples from NPC patients

**Figure 1:**
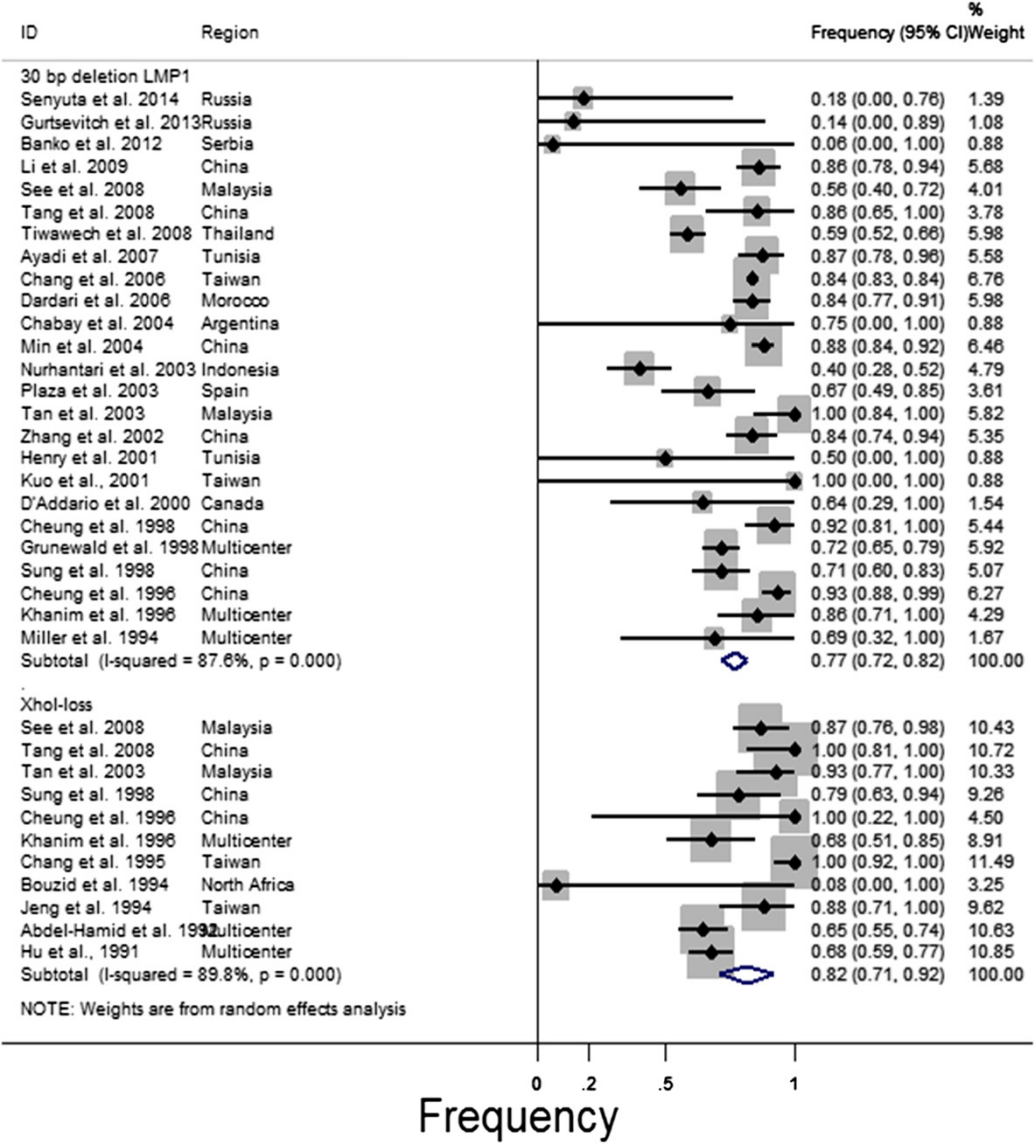
Forest plot of the frequency of the occurrence of the two outcomes of interest in the patients with NPC. The confidence interval (CI) was 95%, and the diamond represents the pooled estimate. ID = identification of study.

The estimated frequencies of the EBV LMP1 variants in the samples from the NPC patients were determined and are shown in Figure 1. Thus, the estimated pooled frequency of 30-bp del-LMP1 was 77% (95% CI: 72 to 82, *P* = 0.00). For the control groups of the healthy controls and pathological samples, the estimated pooled frequency of 30-bp del-LMP1 was 46% (95% CI: 33 to 58, *P* = 0.00) and 39% (95% CI: 31 to 47, *P* = 0.49) (Additional file 5), respectively, that is, both of the control group frequencies were lower than those in the samples from the NPC patients.

For the XhoI-loss variable, the estimated pooled frequency was 82% (95% CI: 71 to 92, *P* = 0.00) in the NPC patients (see Figure 1). For the healthy and pathologic groups, the pooled rate was 62% (95% CI: 33 to 91, *P* = 0.002) and 76% (95% CI: 68 to 84, *P* = 0.3), respectively (Additional file 5).

The estimated pooled frequency was 79% (95% CI: 73 to 85, *P* = 0.00) for 30-bp del-LMP1 in the NPC patients found in Asia, which was the largest among the regions studied. The second highest frequency was observed in America, with a pooled frequency of 64% (95% CI: 35 to 93, *P* = 0.78). Finally, the third highest pooled frequency was 59% (95% CI: 45 to 73, *P* = 0.00), which was observed in Europe and North Africa. Similarly, the pooled frequency of XhoI-loss in individuals with NPC was higher for studies conducted in Asia (92%, 95% CI: 87 to 98, *P* = 0.00), followed by America (29%, 95% CI: 0 to 100) and by the regions from Europe and North Africa (9%, 95% CI: 0 to 44) (Additional file 6).

### A meta-analysis of the association between the 30-bp del-LMP1 and XhoI-loss with NPC susceptibility

**Figure 2:**
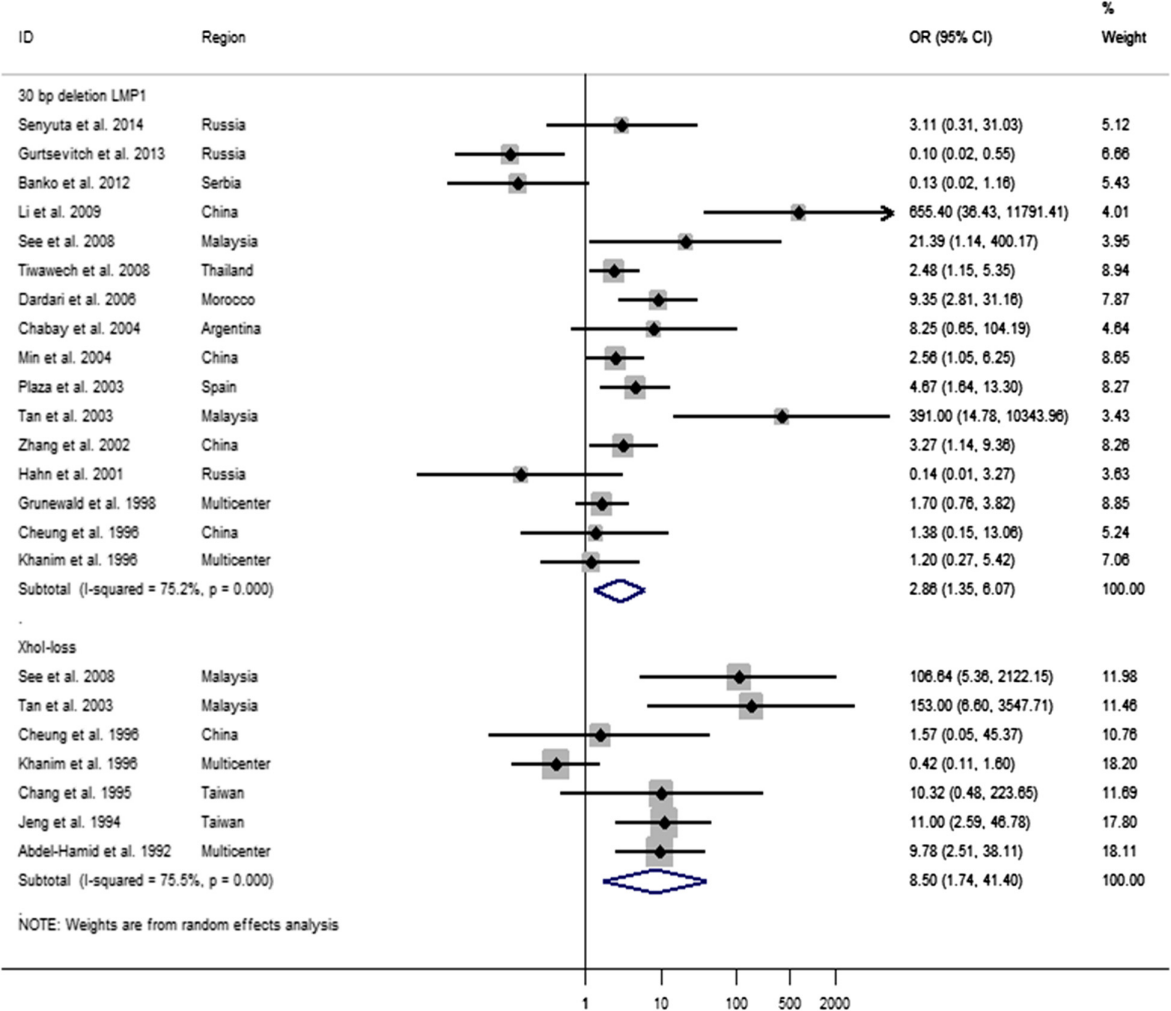
Forest plot of the OR for the 30-bp del-LMP1 and XhoI-loss in NPC. The diamond represents the pooled OR and its 95% confidence intervals (CIs). ID = identification of study.

The OR, known as an association test, was calculated to assess the relationship between the LMP1 variants and NPC. Thus, there was a significant association between the 30-bp del-LMP1/XhoI-loss and NPC susceptibility (30-bp del-LMP1: OR = 2.86, 95% CI = 1.35 to 6.07, *P* = 0.00; XhoI-loss: OR = 8.5, 95% CI = 1.7 to 41, *P* = 0.00) (Figure 2). However, when we analyzed the association of the occurrence of the simultaneous outcome, that is, the co-existence of the 30-bp del-LMP1 and XhoI-loss with the susceptibility to NPC, there was not a significant association (OR = 1.09, 95% CI: 0.06 to 18.79, *P* = 0.002) (data not shown). However, there was significant heterogeneity, and due to this factor, the studies were analyzed in subgroups.

#### The histological subsets of the NPC and LMP1 EBV variants

According to the World Health Organization (WHO), NPC can be classified into three types according to the degree of histopathological differentiation, which include type I (keratinizing squamous cells carcinoma), type IIa (non-keratinizing, previously classified as type II), and type IIb (undifferentiated carcinoma, previously classified as type III) [65]. Therefore, when the selected studies reported this information, they were divided according to the histopathological classification to assess the association between the types of NPC and the EBV LMP1 variants. Thus, a significant association was found between the occurrence of 30-bp del-LMP1 with type III NPC (OR = 2.6, 95% CI: 1.12 to 6.03, *P* = 0.006) and also with type I/II NPC (OR = 2.65, 95% CI: 1.45 to 4.85, *P* = 0.7). Similarly, there was a significant association between type III NPC and XhoI-loss (OR = 65, 95% CI: 1.8 to 2,369, *P* = 0.1); likewise there was an association for types I/II NPC and XhoI-loss (OR = 13.67, 95% CI: 3.12 to 60, *P* = 0.3) (Additional file 7).

#### The association between the EBV LMP1 variants and the susceptibility to NPC by study region

**Figure 3:**
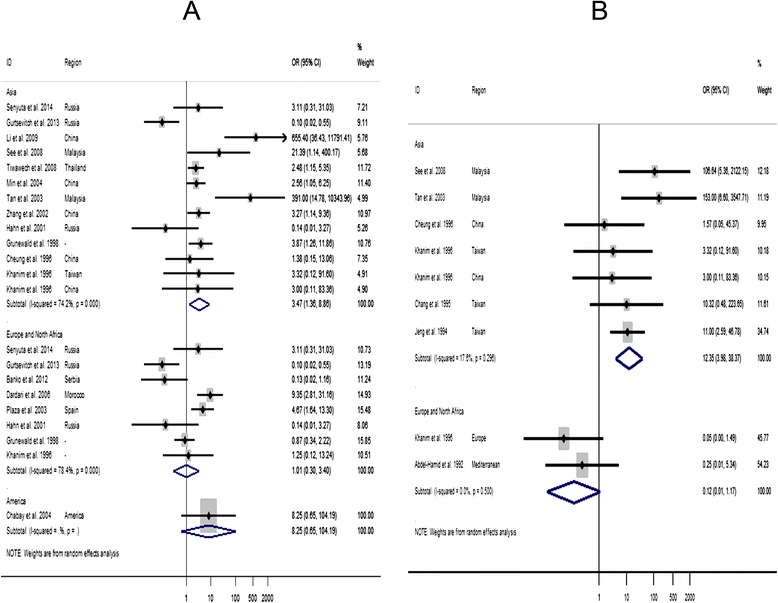
Forest plot of the odds ratio (OR) in relation to the origin from the studies for the outcome of the 30-bp del-LMP1 **(A)** and XhoI-loss **(B)** in patients with NPC. CI = confidence interval; ID = identification of study.

A significant association between 30-bp del-LMP1 and the susceptibility to NPC was found in the studies conducted in Asia (OR = 3.47, 95% CI: 1.36 to 8.86, *P* = 0.00), while for the studies conducted in Europe and North Africa, there was no association (OR = 1.01, 95% CI: 0.3 to 3.4, *P* = 0.00) (Figure 3A). Similarly, there was a higher association between XhoI-loss and NPC susceptibility for studies conducted in Asia (OR = 12.35, 95% CI: 3.98 to 38, *P* = 0.27). For the studies conducted in Europe and North Africa, there was no association between XhoI-loss and NPC susceptibility because the pooled OR was 0.12 (95% CI: 0.01 to 1.17, *P* = 0.5) (Figure 3B).

### Sensitivity analysis and publication bias

The studies that presented results considered discordant were temporarily removed to assess their individual influences on the pooled ORs. The individual removal did not significantly affect the pooled OR. However, due to the large variation of the ORs between 30-bp del-LMP1 and the susceptibility to NPC found in the studies of Li et al*.* [38] and Tan et al*.* [49], the ORs were removed, and the pooled OR was reduced by 31%, reaching 2.86 (95% CI: 1.35 to 6.07) to 1.96 (95% CI: 1.05 to 3.66, *P* = 0.001). Similarly for XhoI-loss, the See et al*.* [39] and Tan et al*.* [49] studies exhibited an OR very different from the other studies, and when both were removed, the pooled OR was reduced by 8.5 and equaled 3.69 (95% CI: 0.75 to 18, *P* = 0.005). However, removing these studies did not reduce the significant heterogeneity found. Therefore, the reduction of heterogeneity observed in the studies that analyzed 30-bp del-LMP1 occurred when the following studies were removed: Senyuta et al*.* [35], Gurtsevitch et al*.* [36], Banko et al*.* [37], Li et al*.* [38], and Hahn et al*.* [51]. In this case, the heterogeneity was reduced to 48% (*P* = 0.036) with an OR of 3.51 (95% CI: 2 to 6) (data not shown).

For the studies that analyzed XhoI-loss, only the study conducted by Khanim et al*.* [29] was found to cause heterogeneity, and when it was removed, the heterogeneity decreased to 19% (*P* = 0.28) with a pooled OR of 16.83 (95% CI: 6 to 43).

For the analysis of the publication bias, a funnel plot was used, and because it was symmetrical, it indicated that the existence of a selection bias was unlikely (Additional file 8). Additionally, by Egger’s test, there was also no sign of the occurrence of a selection bias for the studies included in the estimate of the OR and for the studies that analyzed the outcome of 30-bp del-LMP1 (coefficient −1.22, 95% CI: −4 to 1.64, *P* value 0.36) or XhoI-loss (coefficient −8.2, 95% CI: −248 to 231, *P* value 0.7) in the biopsies from the NPC patients. Additionally, regarding the calculation of the frequency, there was no sign of publication bias by the funnel plot and by Egger’s test (30-bp del-LMP1, coefficient −0.9, 95% CI: −2 to 0.2, *P* value 0.1; XhoI, coefficient 0.2, 95% CI: −2.8 to 3.2, *P* value 0.9).

## Discussion

This meta-analysis provided evidence that both the 30-bp deletion and XhoI-loss (EBV LMP1 gene) were more frequently found in the samples from patients with NPC than in the control groups. In this case, 30-bp del-LMP1 was 1.5 times higher in the NPC patients than in the control group. While the outcome of XhoI-loss was only 1.15 times higher in the NPC patients than in the control group, the simultaneous occurrence of the outcomes of interest (the co-existence of the 30-bp del-LMP1 and XhoI-loss), unlike our previous data, showed no association (OR = 1.9, 95% CI: 0.06 to 18.79), although this result has been found by a few studies. This result shows that LMP1 variants occur with a considerable frequency in healthy subjects; therefore, there are many other non-viral factors involved in the etiopathogenesis of NPC. Thus, it is known that the development of NPC is multifactorial, involving the existence of a genetic predisposition of the individual, along with environmental factors [66]. However, one of the factors that has been most often correlated with the risk of NPC is the viral factor.

Because the etiology of NPC is complex, other meta-analyses have emerged to analyze the involvement of various factors in the susceptibility to NPC. In this sense, Xue et al*.* [67] conducted a meta-analysis of cohort and case–control studies to establish the relationship between smoking and the risk of NPC. This analysis showed that individuals who smoked always had a 60% greater probability of risk for NPC compared to that of individuals who have never smoked. Regarding genetic predisposition, studies have found an association between glutathione S-transferase M1 (GSTM1) and glutathione S-transferase T1 (GSTT1) with an increased risk for the development of NPC [67,68]. Also, the role of human leukocyte antigen (HLA) in the emergence of NPC has been examined, and some specific haplotypes and/or alleles from the HLA region have been associated with NPC [69-71]. For this reason, studies have found a high prevalence of HLA allele patterns found in endemic NPC regions, for example, HLA-A2 and HLA-Bw46 [72,73], which were associated with an increased risk for the development of NPC. These results have shown that many genes may contribute to an increased susceptibility to NPC, and some interesting studies show that there is a pattern of distribution of the HLA haplotypes and/or alleles that vary in different geographical areas characterized by differences in the prevalence of NPC [72-75].

LMP1 is considered an oncogenic product from EBV, and although its expression has been positively correlated with metastasis in NPC [76], its etiopathogenesis remains poorly understood. In this context, authors have postulated that variants of LMP1 exist with greater tumorigenic potential, such as the specific 30-bp deletion at the C-terminal region and the loss of the restriction site at the N-terminus (XhoI), which were our target outcomes of interest. Consequently, our meta-analysis has found that there was a positive association, but we postulated that at this moment, this outcome alone does not explain the risk of the emergence and development of NPC because these LMP1 variants were also found with a considerable frequency in the control groups. Similarly, these LMP1 variants were found in other cancers, such as Burkitt’s lymphoma [77], gastric carcinoma [78], and Hodgkin’s lymphoma [79,80], and also in the Japanese population [81]. With this background, the two LMP1 variants cannot be considered specific markers of NPC but could be considered the predominant variants in this type of cancer.

One factor that always draws attention in NPC is its remarkable geographical distribution because, while NPC is rare in most of the world, for the Southeast Asian region, especially in the South China region, this type of cancer is endemic. For example, the incidence of NPC in some provinces of Southern China is up to 30 times higher than in the rest of the world [82]. Thus, the question inevitably arises: what are the factors that explain the higher incidence of NPC in these endemic regions? Initially, the answer was related to an increased susceptibility of the people of these regions with the 30-bp del-LMP1 variant, as this is a variant commonly found in NPC biopsies. However, Zhang et al*.* [50] found similar levels of this LMP1 polymorphism among samples from endemic and non-endemic regions in South and North China, respectively. Therefore, the LMP1 variant failed to explain the high incidence of NPC in China, showing that there are other complex factors. Although these regions are composed of a very similar ethnic group, most likely, there are some genetic differences among this population. Accordingly, Wang et al*.* [83] found a higher frequency of HLA-A (*)30 in healthy individuals from the North China region, which has been associated with NPC resistance. Additionally, Ren et al*.* [84] found that the risk of developing NPC increases with a family history of the disease, meaning that individuals in South China who have a greater family history and incidence of NPC consequently have a greater susceptibility to NPC.

As previously mentioned, although EBV is associated with NPC, it most likely is not the only factor that would justify the high incidence of NPC in some parts of the world. A set of factors could contribute to the emergence of NPC, such as the type of diet, especially considering that multiple dietary factors, such as a high consumption of salted fish and eggs have been associated with the risk of NPC [85]. In addition, several authors have consistently found an associated risk between NPC and individuals who often feed on salt-preserved fish. These data were obtained from regions endemic for NPC, where this type of food is traditional for the people who live in these places [86,87].

This is the first meta-analysis that evaluated an association between the 30-bp deletion and XhoI-loss (EBV LMP1 gene) on the risk of NPC. Our results suggest an association between the outcomes of interest. However, we recognize that there were some limitations in our study. First, there was significant heterogeneity in the results; thus, a sensitivity analysis and subgroups showed that the types of controls and histologic types from NPC caused heterogeneity, but we did cannot determine if other variables such as the sample size or the origin of the studies caused the heterogeneity. Second, the loss of the original data from the reviewed studies limited our evaluation of other potential interactions, such as survival analysis and the stage of NPC associated with the LMP1 variants. Third, the number of studies included in the analysis was high, but the sample sizes included in the studies were modest. This factor may reduce the statistical power of the association between the outcome of interest and the risk of cancer. Fourth, articles in the Chinese language were not included; consequently, there is a possibility of a selection bias, even with the funnel plot and Egger’s test indicating no significant publication bias. Finally, our results of the ORs are based on unadjusted estimates, which may suffer from a confounding bias; therefore, a more precise analysis is required and must be conducted and adjusted for other factors jointly, such as cancer stage, age, sex, and genetic and environmental factors involved.

## Conclusions

Our results suggest an association between the 30-bp del-LMP1 and XhoI-loss with NPC susceptibility. However, our data should be interpreted with caution because the sample size was small, and there was heterogeneity between the studies. For sensitivity analysis, individual removal did not significantly affect heterogeneity and the pooled ORs. In summary, further studies with a larger number of individuals will be required to measure the overall joint association of the various factors that might be involved in the triggering of NPC.

## Additional files

Additional file 1:
**PRISMA Checklist.** PRISMA 2009 checklist showing guidelines for systematic reviews and meta-analysis. Additional file 2:
**PRISMA flow diagram.** PRISMA 2009 flow diagram of the cohort and case–control studies included in the meta-analysis. Additional file 3:
**List of the pre-selected studies.** List of the pre-selected studies that were excluded due to the appropriate justifications. Additional file 4:
**Methodologic quality of cohort and case–control studies.** Methodologic quality of cohort and case–control studies included in the meta-analysis. Additional file 5:
**Forest plot of the frequency of the 30-bp del-LMP1 and XhoI-loss.** Forest plot of the frequency of the 30-bp del-LMP1 and XhoI-loss in the control groups. Additional file 6:
**Forest plot of the frequency of the 30-bp del-LMP1 and XhoI-loss.** Forest plot of the frequency of the 30-bp del-LMP1 (left) and XhoI-loss (right) in relation to the origin from the studies. Additional file 7:
**Forest plot of the OR regarding the histological type of NPC.** Forest plot of the OR regarding the histological type of NPC associated with the 30-bp del-LMP1 (left) and XhoI-loss (right). Additional file 8:
**Funnel plot for publication bias.** Funnel plot for publication bias of the 30-bp del-LMP1 and Xho-loss.

## Abbreviations

CTAR: C-terminal activation regions
EBV: Epstein-Barr virus
GSTM1: Glutathione S-transferase M1
GSTT1: Glutathione S-transferase T1
HLA: Human leukocyte antigen
LMP: Latent membrane protein
NOS: Newcastle-Ottawa Scale
NPC: Nasopharyngeal carcinoma
PRISMA: Preferred Reporting Items for Systematic Reviews and Meta-Analyses
