# Supplementary material for: The Epstein-Barr virus latent membrane protein-1 (LMP1) 30-bp deletion and XhoI-polymorphism in nasopharyngeal carcinoma: a meta-analysis of observational studies
Source: Syst Rev. 2015 Apr 13;4:46. doi: 10.1186/s13643-015-0037-z (PMC4404015; doi:10.1186/s13643-015-0037-z)
Supplement: Additional file 2: — PRISMA flow diagram. PRISMA 2009 flow diagram of the cohort and case–control studies included in the meta-analysis. [file 13643_2015_37_MOESM2_ESM.doc]

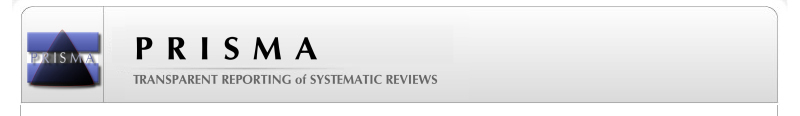
**PRISMA 2009 Flow Diagram**

**Screening**

**Included**

**Eligibility**

**Identification**

Records identified through database searching (n = 1280)

PubMed=398

SciELO=21

ScienceDirect=861

Additional records identified through other sources
(n = 23)

Records after duplicates removed
(n = 1224)

Records screened
(n = 1224)

Records excluded after abstract review
(n = 1149)

Full-text articles assessed for eligibility
(n = 75 )

Full-text articles excluded, with reasons
(n = 44)

Studies included in qualitative synthesis
(n = 31)

Studies included in quantitative synthesis (meta-analysis)
(n = 31)
