# Supplementary material for: The Epstein-Barr virus latent membrane protein-1 (LMP1) 30-bp deletion and XhoI-polymorphism in nasopharyngeal carcinoma: a meta-analysis of observational studies
Source: Syst Rev. 2015 Apr 13;4:46. doi: 10.1186/s13643-015-0037-z (PMC4404015; doi:10.1186/s13643-015-0037-z)
Supplement: Additional file 3: — List of the pre-selected studies. List of the pre-selected studies that were excluded due to the appropriate justifications. [file 13643_2015_37_MOESM3_ESM.docx]

**Additional file 3: Table 2 of excluded studies during the analysis regarding inclusion process of meta-analysis**

| **Study** | **Journal** | **Year** | **Reasons for exclusion** |
| --- | --- | --- | --- |
| [Berger C](http://www.ncbi.nlm.nih.gov/pubmed?term=Berger%20C%5BAuthor%5D&cauthor=true&cauthor_uid=9359741), [McQuain C](http://www.ncbi.nlm.nih.gov/pubmed?term=McQuain%20C%5BAuthor%5D&cauthor=true&cauthor_uid=9359741), [Sullivan JL](http://www.ncbi.nlm.nih.gov/pubmed?term=Sullivan%20JL%5BAuthor%5D&cauthor=true&cauthor_uid=9359741), et al. **The 30-bp deletion variant of Epstein-Barr virus–encoded latent membrane protein-1 prevails in acute infectious mononucleosis.** | The Journal of Infectious Diseases | 1997 | Non-NPC |
| [Tsuji A](http://www.ncbi.nlm.nih.gov/pubmed?term=Tsuji%20A%5BAuthor%5D&cauthor=true&cauthor_uid=18765528), [Wakisaka N](http://www.ncbi.nlm.nih.gov/pubmed?term=Wakisaka%20N%5BAuthor%5D&cauthor=true&cauthor_uid=18765528), [Kondo S](http://www.ncbi.nlm.nih.gov/pubmed?term=Kondo%20S%5BAuthor%5D&cauthor=true&cauthor_uid=18765528), et al. **Induction of receptor for advanced glycation end products by EBV latent membrane protein 1and its correlation with angiogenesis and cervical lymph node metastasis in nasopharyngeal carcinoma** | Clinical Cancer Research | 2008 | Non-30-bp deletion EBV LMP1 |
| [Knecht H](http://www.ncbi.nlm.nih.gov/pubmed?term=Knecht%20H%5BAuthor%5D&cauthor=true&cauthor_uid=8219183), [Bachmann E](http://www.ncbi.nlm.nih.gov/pubmed?term=Bachmann%20E%5BAuthor%5D&cauthor=true&cauthor_uid=8219183), [Brousset P](http://www.ncbi.nlm.nih.gov/pubmed?term=Brousset%20P%5BAuthor%5D&cauthor=true&cauthor_uid=8219183), et al. **Deletions within the LMP1 oncogene of Epstein-Barr virus are clustered in Hodgkin's disease and identical to those observed in nasopharyngeal carcinoma.** | Blood Journal | 1993 | Non-NPC |
| [Lo AK](http://www.ncbi.nlm.nih.gov/pubmed?term=Lo%20AK%5BAuthor%5D&cauthor=true&cauthor_uid=12746479)^1^, [Liu Y](http://www.ncbi.nlm.nih.gov/pubmed?term=Liu%20Y%5BAuthor%5D&cauthor=true&cauthor_uid=12746479), [Wang XH](http://www.ncbi.nlm.nih.gov/pubmed?term=Wang%20XH%5BAuthor%5D&cauthor=true&cauthor_uid=12746479), et al. **Alterations of biologic properties and gene expression in nasopharyngeal epithelial cells by the Epstein-Barr virus–encoded latent membrane protein 1.** | Laboratory investigation; a Journal of technical methods and pathology | 2003 | Non-30-bp deletion EBV LMP1 |
| [Sandvej K](http://www.ncbi.nlm.nih.gov/pubmed?term=Sandvej%20K%5BAuthor%5D&cauthor=true&cauthor_uid=7994023), [Peh SC](http://www.ncbi.nlm.nih.gov/pubmed?term=Peh%20SC%5BAuthor%5D&cauthor=true&cauthor_uid=7994023), [Andresen BS](http://www.ncbi.nlm.nih.gov/pubmed?term=Andresen%20BS%5BAuthor%5D&cauthor=true&cauthor_uid=7994023), et al. **Identification of potential hot spots in the carboxy-terminal part of the Epstein-Barr virus (EBV) BNLF-1 gene in both malignant and benign EBV-associated diseases: high frequency of a 30-bp deletion in Malaysian and Danish peripheral T-cell lymphomas.** | Blood Journal | 1994 | Non-NPC |
| [Chiang AK](http://www.ncbi.nlm.nih.gov/pubmed?term=Chiang%20AK%5BAuthor%5D&cauthor=true&cauthor_uid=9935174), [Wong KY](http://www.ncbi.nlm.nih.gov/pubmed?term=Wong%20KY%5BAuthor%5D&cauthor=true&cauthor_uid=9935174), [Liang AC](http://www.ncbi.nlm.nih.gov/pubmed?term=Liang%20AC%5BAuthor%5D&cauthor=true&cauthor_uid=9935174), et al. **Comparative analysis of Epstein-Barr virus gene polymorphisms in nasal T/NK-cell lymphomas and normal nasal tissues: implications on virus strain selection in malignancy.** | International Journal of Cancer | 1999 | Not specified NPC |
| [Bouzid M](http://www.ncbi.nlm.nih.gov/pubmed?term=Bouzid%20M%5BAuthor%5D&cauthor=true&cauthor_uid=9650553), [Sheng W](http://www.ncbi.nlm.nih.gov/pubmed?term=Sheng%20W%5BAuthor%5D&cauthor=true&cauthor_uid=9650553), [Buisson M](http://www.ncbi.nlm.nih.gov/pubmed?term=Buisson%20M%5BAuthor%5D&cauthor=true&cauthor_uid=9650553), et al. **Different distribution of H1-H2 Epstein-Barr virus variant in oropharyngeal virus and in biopsies of Hodgkin’s disease and in nasopharyngeal carcinoma from Algeria.** | International Journal of Cancer | 1998 | Not released 30-bp deletion and XhoI-loss |
| [Chen WG](http://www.ncbi.nlm.nih.gov/pubmed?term=Chen%20WG%5BAuthor%5D&cauthor=true&cauthor_uid=8546204), [Chen YY](http://www.ncbi.nlm.nih.gov/pubmed?term=Chen%20YY%5BAuthor%5D&cauthor=true&cauthor_uid=8546204), [Bacchi MM](http://www.ncbi.nlm.nih.gov/pubmed?term=Bacchi%20MM%5BAuthor%5D&cauthor=true&cauthor_uid=8546204), et al. **Genotyping of Epstein-Barr virus in Brazilian Burkitt's lymphoma and reactive lymphoid tissue. Type A with a high prevalence of deletions within the latent membrane protein gene.** | The American Journal of pathology | 1996 | Non-NPC |
| [Dawson CW](http://www.ncbi.nlm.nih.gov/pubmed?term=Dawson%20CW%5BAuthor%5D&cauthor=true&cauthor_uid=10873763), [Eliopoulos AG](http://www.ncbi.nlm.nih.gov/pubmed?term=Eliopoulos%20AG%5BAuthor%5D&cauthor=true&cauthor_uid=10873763), [Blake SM](http://www.ncbi.nlm.nih.gov/pubmed?term=Blake%20SM%5BAuthor%5D&cauthor=true&cauthor_uid=10873763), et al. **Identification of functional differences between prototype Epstein-Barr virus-encoded LMP1 and a nasopharyngeal carcinoma-derived LMP1 in human epithelial cells.** | Virology | 2000 | Analysis in vitro |
| [Scheinfeld AG](http://www.ncbi.nlm.nih.gov/pubmed?term=Scheinfeld%20AG%5BAuthor%5D&cauthor=true&cauthor_uid=9284829), [Nador RG](http://www.ncbi.nlm.nih.gov/pubmed?term=Nador%20RG%5BAuthor%5D&cauthor=true&cauthor_uid=9284829), [Cesarman E](http://www.ncbi.nlm.nih.gov/pubmed?term=Cesarman%20E%5BAuthor%5D&cauthor=true&cauthor_uid=9284829), et al. **Epstein-Barr virus latent membrane protein-1 oncogene deletion in post-transplantation lymphoproliferative disorders.** | The American Journal of pathology | 1997 | Did not specify the NPC |
| [Abdelmajid Khabir](http://www.ncbi.nlm.nih.gov/pubmed/?term=Khabir%20A%5Bauth%5D), [Hela Karray](http://www.ncbi.nlm.nih.gov/pubmed/?term=Karray%20H%5Bauth%5D), [Sandrine Rodriguez](http://www.ncbi.nlm.nih.gov/pubmed/?term=Rodriguez%20S%5Bauth%5D), et al. **EBV latent membrane protein 1 abundance correlates with patient age but not with metastatic behavior in North African nasopharyngeal carcinomas.** | Virology Journal | 2005 | Not analyzed 30-bp deletion |
| [Johnson RJ](http://www.ncbi.nlm.nih.gov/pubmed?term=Johnson%20RJ%5BAuthor%5D&cauthor=true&cauthor_uid=9557692), [Stack M](http://www.ncbi.nlm.nih.gov/pubmed?term=Stack%20M%5BAuthor%5D&cauthor=true&cauthor_uid=9557692), [Hazlewood SA](http://www.ncbi.nlm.nih.gov/pubmed?term=Hazlewood%20SA%5BAuthor%5D&cauthor=true&cauthor_uid=9557692), et al. **The 30-Base-Pair Deletion in Chinese Variants of the Epstein-Barr Virus LMP1 Gene Is Not the Major Effector of Functional Differences between Variant LMP1 Genes in Human Lymphocytes.** | Journal of Virology | 1998 | Analysis in vitro |
| Kim LH and Peh SC. **Epstein-Barr virus-associated lymphomas in Malaysia: high frequency of a 30-bp deletion in the viral latent membrane protein-1 (LMP-1) oncogene.** | Journal of Clinical and Experimental Hematopathology | 2003 | Not specified NPC |
| [Kuo TT](http://www.ncbi.nlm.nih.gov/pubmed?term=Kuo%20TT%5BAuthor%5D&cauthor=true&cauthor_uid=15494863), [Shih LY](http://www.ncbi.nlm.nih.gov/pubmed?term=Shih%20LY%5BAuthor%5D&cauthor=true&cauthor_uid=15494863), [Tsang NM](http://www.ncbi.nlm.nih.gov/pubmed?term=Tsang%20NM%5BAuthor%5D&cauthor=true&cauthor_uid=15494863). Nasal NK/T cell lymphoma in Taiwan: a clinicopathologic study of 22 cases, with analysis of histologic subtypes, Epstein-Barr virus LMP-1 gene association, and treatment modalities. | International Journal of surgical pathology | 2004 | Non-NPC |
| Abdulamir AS, Hafidh RR, Abdulmuhaimen N, et al. **The distinctive profile of risk factors of nasopharyngeal carcinoma in comparison with other head and neck cancer types.** | BMC Public Health | 2008 | Not analyzed LMP-1 gene |
| [Dolcetti R](http://www.ncbi.nlm.nih.gov/pubmed?term=Dolcetti%20R%5BAuthor%5D&cauthor=true&cauthor_uid=9057656), [Zancai P](http://www.ncbi.nlm.nih.gov/pubmed?term=Zancai%20P%5BAuthor%5D&cauthor=true&cauthor_uid=9057656), [De Re V](http://www.ncbi.nlm.nih.gov/pubmed?term=De%20Re%20V%5BAuthor%5D&cauthor=true&cauthor_uid=9057656), et al. **Epstein-Barr virus strains with latent membrane protein-1 deletions: prevalence in the Italian population and high association with human immunodeficiency virus –related Hodgkin’s disease.** | Blood Journal | 1997 | Non-NPC |
| [Lorenzetti MA](http://www.ncbi.nlm.nih.gov/pubmed?term=Lorenzetti%20MA%5BAuthor%5D&cauthor=true&cauthor_uid=22205789), [Gantuz M](http://www.ncbi.nlm.nih.gov/pubmed?term=Gantuz%20M%5BAuthor%5D&cauthor=true&cauthor_uid=22205789), [Altcheh J](http://www.ncbi.nlm.nih.gov/pubmed?term=Altcheh%20J%5BAuthor%5D&cauthor=true&cauthor_uid=22205789), et al. **Distinctive Epstein-Barr virus variants associated with benign and malignant pediatric pathologies: LMP1 sequence characterization and linkage with other viral gene polymorphisms.** | Journal of clinical microbiology | 2012 | Non-NPC |
| **Wu-zhong J, Su-ping Z, Yu-ping L, et al.** **Expressions of LMP1 and VEGF proteins in nasopharyngeal carcinoma.** | Chinese Journal of Otorhinolaryngology | 2007 | Not accessible language (article in Chinese) |
| [Tsang NM](http://www.ncbi.nlm.nih.gov/pubmed?term=Tsang%20NM%5BAuthor%5D&cauthor=true&cauthor_uid=12782821) , [Chang KP](http://www.ncbi.nlm.nih.gov/pubmed?term=Chang%20KP%5BAuthor%5D&cauthor=true&cauthor_uid=12782821), [Lin SY](http://www.ncbi.nlm.nih.gov/pubmed?term=Lin%20SY%5BAuthor%5D&cauthor=true&cauthor_uid=12782821), et al. **Detection of Epstein-Barr virus-derived latent membrane protein-1 gene in various head and neck cancers: is it specific for nasopharyngeal carcinoma.** | The Laryngoscope | 2003 | Not analyzed 30-bp deletion |
| [Ou XB](http://www.ncbi.nlm.nih.gov/pubmed?term=Ou%20XB%5BAuthor%5D&cauthor=true&cauthor_uid=18710612), [Chen XY](http://www.ncbi.nlm.nih.gov/pubmed?term=Chen%20XY%5BAuthor%5D&cauthor=true&cauthor_uid=18710612), [Wu MH](http://www.ncbi.nlm.nih.gov/pubmed?term=Wu%20MH%5BAuthor%5D&cauthor=true&cauthor_uid=18710612), et al. **Effects of Epstein-Barr virus latent membrane protein 1 on metastasis of human nasopharyngeal carcinoma cell lines.** | Ai zheng Chinese Journal of cancer | 2008 | Not analyzed 30-bp deletion |
| [Li SN](http://www.ncbi.nlm.nih.gov/pubmed?term=Li%20SN%5BAuthor%5D&cauthor=true&cauthor_uid=8668338), [Chang YS](http://www.ncbi.nlm.nih.gov/pubmed?term=Chang%20YS%5BAuthor%5D&cauthor=true&cauthor_uid=8668338), [Liu ST](http://www.ncbi.nlm.nih.gov/pubmed?term=Liu%20ST%5BAuthor%5D&cauthor=true&cauthor_uid=8668338). **Effect of a 10-amino acid deletion on the oncogenic activity of latent membrane protein 1 of Epstein-Barr virus.** | Oncogene | 1996 | Analysis in vitro |
| [Itakura O](http://www.ncbi.nlm.nih.gov/pubmed?term=Itakura%20O%5BAuthor%5D&cauthor=true&cauthor_uid=8875994), [Yamada S](http://www.ncbi.nlm.nih.gov/pubmed?term=Yamada%20S%5BAuthor%5D&cauthor=true&cauthor_uid=8875994), [Narita M](http://www.ncbi.nlm.nih.gov/pubmed?term=Narita%20M%5BAuthor%5D&cauthor=true&cauthor_uid=8875994), et al. **High prevalence of a 30-base pair deletion and single-base mutations within the carboxy terminal end of the LMP-1 oncogene of Epstein-Barr virus in the Japanese population.** | Oncogene | 1996 | Non-NPC |
| [Plaza G](http://www.ncbi.nlm.nih.gov/pubmed?term=Plaza%20G%5BAuthor%5D&cauthor=true&cauthor_uid=11913680), [Manzanal AI](http://www.ncbi.nlm.nih.gov/pubmed?term=Manzanal%20AI%5BAuthor%5D&cauthor=true&cauthor_uid=11913680), [Fogué L](http://www.ncbi.nlm.nih.gov/pubmed?term=Fogu%C3%A9%20L%5BAuthor%5D&cauthor=true&cauthor_uid=11913680), et al. **Association of Epstein-Barr virus and nasopharyngeal carcinoma in Caucasian patients.** | The Annals of otology, rhinology, and laryngology | 2002 | Not analyzed 30-bp deletion |
| [Hu LF](http://www.ncbi.nlm.nih.gov/pubmed?term=Hu%20LF%5BAuthor%5D&cauthor=true&cauthor_uid=7640034)^1^, [Chen F](http://www.ncbi.nlm.nih.gov/pubmed?term=Chen%20F%5BAuthor%5D&cauthor=true&cauthor_uid=7640034), [Zhen QF](http://www.ncbi.nlm.nih.gov/pubmed?term=Zhen%20QF%5BAuthor%5D&cauthor=true&cauthor_uid=7640034), et al. **Differences in the growth pattern and clinical course of EBV-LMP1 expressing and non-expressing nasopharyngeal carcinomas.** | European Journal of Cancer | 1995 | Not analyzed 30-bp deletion |
| [Suzumiya J](http://www.ncbi.nlm.nih.gov/pubmed?term=Suzumiya%20J%5BAuthor%5D&cauthor=true&cauthor_uid=10609794), [Ohshima K](http://www.ncbi.nlm.nih.gov/pubmed?term=Ohshima%20K%5BAuthor%5D&cauthor=true&cauthor_uid=10609794), [Takeshita M](http://www.ncbi.nlm.nih.gov/pubmed?term=Takeshita%20M%5BAuthor%5D&cauthor=true&cauthor_uid=10609794), et al. **Nasal lymphomas in Japan: a high prevalence of Epstein-Barr virus type A and deletion within the latent membrane protein gene.** | Leukemia e lymphoma | 1999 | Non-NPC |
| [Chen ML](http://www.ncbi.nlm.nih.gov/pubmed?term=Chen%20ML%5BAuthor%5D&cauthor=true&cauthor_uid=1331932), [Tsai CN](http://www.ncbi.nlm.nih.gov/pubmed?term=Tsai%20CN%5BAuthor%5D&cauthor=true&cauthor_uid=1331932), [Liang CL](http://www.ncbi.nlm.nih.gov/pubmed?term=Liang%20CL%5BAuthor%5D&cauthor=true&cauthor_uid=1331932), et al. **Cloning and characterization of the latent membrane protein (LMP) of a specific Epstein-Barr virus variant derived from the nasopharyngeal carcinoma in the Taiwanese population.** | Oncogene | 1992 | Unavailable |
| [Ai J](http://www.ncbi.nlm.nih.gov/pubmed?term=Ai%20J%5BAuthor%5D&cauthor=true&cauthor_uid=22236445), [Xie Z](http://www.ncbi.nlm.nih.gov/pubmed?term=Xie%20Z%5BAuthor%5D&cauthor=true&cauthor_uid=22236445), [Liu C](http://www.ncbi.nlm.nih.gov/pubmed?term=Liu%20C%5BAuthor%5D&cauthor=true&cauthor_uid=22236445), et al. **Analysis of EBNA-1 and LMP-1 variants in diseases associated with EBV infection in Chinese children.** | Virology Journal | 2012 | Non-NPC |
| [Wang X](http://www.ncbi.nlm.nih.gov/pubmed?term=Wang%20X%5BAuthor%5D&cauthor=true&cauthor_uid=23605669), [Wu G](http://www.ncbi.nlm.nih.gov/pubmed?term=Wu%20G%5BAuthor%5D&cauthor=true&cauthor_uid=23605669), [Wang Y](http://www.ncbi.nlm.nih.gov/pubmed?term=Wang%20Y%5BAuthor%5D&cauthor=true&cauthor_uid=23605669), et al. **Gene variations of Epstein-Barr virus nuclear** **antigen 3A in nasopharyngeal carcinomas, gastric carcinomas and healthy carriers in northern China**. | Archives of virology | 2013 | Not analyzed 30-bp deletion |
| [Moumad K](http://www.ncbi.nlm.nih.gov/pubmed?term=Moumad%20K%5BAuthor%5D&cauthor=true&cauthor_uid=23576520), [Lascorz J](http://www.ncbi.nlm.nih.gov/pubmed?term=Lascorz%20J%5BAuthor%5D&cauthor=true&cauthor_uid=23576520), [Bevier M](http://www.ncbi.nlm.nih.gov/pubmed?term=Bevier%20M%5BAuthor%5D&cauthor=true&cauthor_uid=23576520), et al. **Genetic polymorphisms in host innate immune sensor genes and the risk of nasopharyngeal carcinoma in North Africa.** | G3 (Bethesda, Md.) | 2013 | Not analyzed 30-bp deletion and XhoI-loss |
| [Gantuz M](http://www.ncbi.nlm.nih.gov/pubmed?term=Gantuz%20M%5BAuthor%5D&cauthor=true&cauthor_uid=23305886), [Lorenzetti MA](http://www.ncbi.nlm.nih.gov/pubmed?term=Lorenzetti%20MA%5BAuthor%5D&cauthor=true&cauthor_uid=23305886), [Altcheh J](http://www.ncbi.nlm.nih.gov/pubmed?term=Altcheh%20J%5BAuthor%5D&cauthor=true&cauthor_uid=23305886), et al. **LMP1 promoter sequence analysis in Epstein Barr virus pediatric infection reveals preferential circulation of B95.8 related variants in Argentina.** | Infection, genetics and evolution: journal of molecular epidemiology and evolutionary genetics in infectious diseases | 2013 | Not analyzed 30-bp deletion and XhoI-loss |
| [Cui Y](http://www.ncbi.nlm.nih.gov/pubmed?term=Cui%20Y%5BAuthor%5D&cauthor=true&cauthor_uid=20948223), [Wang Y](http://www.ncbi.nlm.nih.gov/pubmed?term=Wang%20Y%5BAuthor%5D&cauthor=true&cauthor_uid=20948223), [Liu X](http://www.ncbi.nlm.nih.gov/pubmed?term=Liu%20X%5BAuthor%5D&cauthor=true&cauthor_uid=20948223), et al. **Genotypic analysis of Epstein-Barr virus isolates associated with nasopharyngeal carcinoma in Northern China.** | Intervirology | 2011 | Not analyzed 30-bp deletion |
| [Saechan V](http://www.ncbi.nlm.nih.gov/pubmed?term=Saechan%20V%5BAuthor%5D&cauthor=true&cauthor_uid=20427564), [Settheetham-Ishida W](http://www.ncbi.nlm.nih.gov/pubmed?term=Settheetham-Ishida%20W%5BAuthor%5D&cauthor=true&cauthor_uid=20427564), [Kimura R](http://www.ncbi.nlm.nih.gov/pubmed?term=Kimura%20R%5BAuthor%5D&cauthor=true&cauthor_uid=20427564), et al. **Epstein–Barr virus strains defined by the latent membrane protein 1 sequence characterize Thai ethnic groups.** | The Journal of general virology | 2010 | Not specified NPC and 30-bp deletion |
| [Wang Y](http://www.ncbi.nlm.nih.gov/pubmed?term=Wang%20Y%5BAuthor%5D&cauthor=true&cauthor_uid=20336720), [Zhang X](http://www.ncbi.nlm.nih.gov/pubmed?term=Zhang%20X%5BAuthor%5D&cauthor=true&cauthor_uid=20336720), [Chao Y](http://www.ncbi.nlm.nih.gov/pubmed?term=Chao%20Y%5BAuthor%5D&cauthor=true&cauthor_uid=20336720), et al. **New variations of Epstein-Barr virus-encoded small RNA genes in nasopharyngeal carcinomas, gastric carcinomas, and healthy donors in northern China.** | Journal of medical virology | 2010 | Not analyzed LMP1 and 30-bp deletion |
| [Klemenc P](http://www.ncbi.nlm.nih.gov/pubmed?term=Klemenc%20P%5BAuthor%5D&cauthor=true&cauthor_uid=16789009), [Marin J](http://www.ncbi.nlm.nih.gov/pubmed?term=Marin%20J%5BAuthor%5D&cauthor=true&cauthor_uid=16789009), [Soba E](http://www.ncbi.nlm.nih.gov/pubmed?term=Soba%20E%5BAuthor%5D&cauthor=true&cauthor_uid=16789009), et al. **Distribution of Epstein-Barr virus genotypes in throat washings, sera, peripheral blood lymphocytes and in EBV positive tumor biopsies from Slovenian patients with nasopharyngeal carcinoma.** | Journal of medical virology | 2006 | Not analyzed LMP1 and 30-bp deletion |
| [Wang Y](http://www.ncbi.nlm.nih.gov/pubmed?term=Wang%20Y%5BAuthor%5D&cauthor=true&cauthor_uid=19941915), [Liu X](http://www.ncbi.nlm.nih.gov/pubmed?term=Liu%20X%5BAuthor%5D&cauthor=true&cauthor_uid=19941915), [Xing X](http://www.ncbi.nlm.nih.gov/pubmed?term=Xing%20X%5BAuthor%5D&cauthor=true&cauthor_uid=19941915), et al. **Variations of Epstein-Barr virus nuclear antigen 1 gene in gastric carcinomas and nasopharyngeal carcinomas from Northern China.** | Virus research | 2010 | Not analyzed LMP1 |
| [Chen JN](http://www.ncbi.nlm.nih.gov/pubmed?term=Chen%20JN%5BAuthor%5D&cauthor=true&cauthor_uid=21723347), [Jiang Y](http://www.ncbi.nlm.nih.gov/pubmed?term=Jiang%20Y%5BAuthor%5D&cauthor=true&cauthor_uid=21723347), [Li HG](http://www.ncbi.nlm.nih.gov/pubmed?term=Li%20HG%5BAuthor%5D&cauthor=true&cauthor_uid=21723347), et al. **Epstein-Barr virus genome polymorphisms of Epstein-Barr virus-associated gastric carcinoma in gastric remnant carcinoma** **in Guangzhou, southern China, an endemic area of nasopharyngeal carcinoma** | Virus research | 2011 | Non-NPC |
| [Nie Y](http://www.ncbi.nlm.nih.gov/pubmed?term=Nie%20Y%5BAuthor%5D&cauthor=true&cauthor_uid=23266831), [Sun Y](http://www.ncbi.nlm.nih.gov/pubmed?term=Sun%20Y%5BAuthor%5D&cauthor=true&cauthor_uid=23266831), [Wang Y](http://www.ncbi.nlm.nih.gov/pubmed?term=Wang%20Y%5BAuthor%5D&cauthor=true&cauthor_uid=23266831), et al. **Epstein-Barr virus gene polymorphism in different parts of the same nasopharyngeal carcinoma patient.** | Archives of virology | 2013 | Not analyzed 30-bp deletion |
| [Kingma DW](http://www.ncbi.nlm.nih.gov/pubmed?term=Kingma%20DW%5BAuthor%5D&cauthor=true&cauthor_uid=8704180), [Weiss WB](http://www.ncbi.nlm.nih.gov/pubmed?term=Weiss%20WB%5BAuthor%5D&cauthor=true&cauthor_uid=8704180), [Jaffe ES](http://www.ncbi.nlm.nih.gov/pubmed?term=Jaffe%20ES%5BAuthor%5D&cauthor=true&cauthor_uid=8704180), et al. **Epstein-Barr virus latent membrane protein-1 oncogene deletions: correlations with malignancy in Epstein-Barr virus--associated lymphoproliferative disorders and malignant lymphomas.** | Blood Journal | 1996 | Non-NPC |
| [Wu G](http://www.ncbi.nlm.nih.gov/pubmed?term=Wu%20G%5BAuthor%5D&cauthor=true&cauthor_uid=22302288), [Wang Y](http://www.ncbi.nlm.nih.gov/pubmed?term=Wang%20Y%5BAuthor%5D&cauthor=true&cauthor_uid=22302288), [Chao Y](http://www.ncbi.nlm.nih.gov/pubmed?term=Chao%20Y%5BAuthor%5D&cauthor=true&cauthor_uid=22302288), et al. **Characterization of Epstein-Barr virus type 1 nuclear antigen 3C sequence patterns of nasopharyngeal and gastric carcinomas in northern China.** | Archives of virology | 2012 | Not analyzed 30-bp deletion |
| [Shu CH](http://www.ncbi.nlm.nih.gov/pubmed?term=Shu%20CH%5BAuthor%5D&cauthor=true&cauthor_uid=10820907), [Tu TY](http://www.ncbi.nlm.nih.gov/pubmed?term=Tu%20TY%5BAuthor%5D&cauthor=true&cauthor_uid=10820907). **Prevalence of the Taiwan variant of the Epstein-Barr virus in nasopharyngeal carcinoma patients and normal individuals.** | Zhonghua yi xue za zhi Chinese Medical Journal | 2000 | Unavailable |
| [Correa RM](http://www.ncbi.nlm.nih.gov/pubmed?term=Correa%20RM%5BAuthor%5D&cauthor=true&cauthor_uid=15221903), [Fellner MD](http://www.ncbi.nlm.nih.gov/pubmed?term=Fellner%20MD%5BAuthor%5D&cauthor=true&cauthor_uid=15221903), [Alonio LV](http://www.ncbi.nlm.nih.gov/pubmed?term=Alonio%20LV%5BAuthor%5D&cauthor=true&cauthor_uid=15221903), et al. **Epstein-barr virus (EBV) in healthy carriers: Distribution of genotypes and 30 bp deletion in latent membrane protein-1 (LMP-1) oncogene.** | Journal medical of virology | 2004 | Non-NPC |
| Zheng MS, Li DJ, Liu QL, et al. **Genomic sequence analysis of Epstein-Barr virus strain GD1 from a nasopharyngeal carcinoma patient.** | Journal of Virology | 2005 | Not reported variables of interest |
| Lin SX, Zong YS, Zhang M, et al. **Study of sequence variations of Epstein-Barr virus LMP1 gene in nasopharyngeal carcinoma**. | Zhonghua Bing Li Xue Za Zhi | 2005 | Article in Chinese language |
| Lin SX, Zong YS, Wu QL, et al. **Loss of an XhoI-site within N**-**terminal region of Epstein-Barr virus LMP1 gene in nasopharyngeal carcinoma.** | Ai Zheng | 2005 | Article in Chinese language |
