# Supplementary material for: The Epstein-Barr virus latent membrane protein-1 (LMP1) 30-bp deletion and XhoI-polymorphism in nasopharyngeal carcinoma: a meta-analysis of observational studies
Source: Syst Rev. 2015 Apr 13;4:46. doi: 10.1186/s13643-015-0037-z (PMC4404015; doi:10.1186/s13643-015-0037-z)
Supplement: Additional file 4: — Methodologic quality of cohort and case–control studies. Methodologic quality of cohort and case–control studies included in the meta-analysis. [file 13643_2015_37_MOESM4_ESM.docx]

**Additional file 4: Methodologic quality of cohort and case-control studies included in the meta-analysis**

Methodologic quality of case-control studies included in the meta-analysis (NOS*)

| Study and year | Adequate definition of cases | Representa-tiveness of cases | Selection of controls | Definition of controls | Controls for important factors | Exposure assessment | Same method of ascertainment for all subjects | Non-response rate | Total quality scores |
| --- | --- | --- | --- | --- | --- | --- | --- | --- | --- |
| Senyuta 2014 | ☆ | ☆ | ☆ | ☆ | - | ☆ | ☆ | ☆ | 7 |
| Gurtsevitch 2013 | ☆ | ☆ | ☆ | ☆ | - | ☆ | ☆ | ☆ | 7 |
| Banko 2012 | ☆ | ☆ | ☆ | ☆ | - | ☆ | ☆ | ☆ | 7 |
| Li 2009 | ☆ | ☆ | ☆ | ☆ | - | ☆ | ☆ | ☆ | 7 |
| See 2008 | ☆ | ☆ | ☆ | ☆ | - | ☆ | ☆ | ☆ | 7 |
| Tiwawech 2008 | ☆ | ☆ | ☆ | ☆ | - | ☆ | ☆ | ☆ | 7 |
| Dardari 2006 | ☆ | ☆ | ☆ | ☆ | - | ☆ | ☆ | ☆ | 7 |
| Chabay 2004 | ☆ | ☆ | ☆ | ☆ | - | ☆ | ☆ | ☆ | 7 |
| Min 2004 | ☆ | ☆ | ☆ | ☆ | - | ☆ | ☆ | ☆ | 7 |
| Plaza 2003 | ☆ | ☆ | ☆ | ☆ | - | ☆ | ☆ | ☆ | 7 |
| Tan 2003 | ☆ | ☆ | ☆ | ☆ | - | ☆ | ☆ | ☆ | 7 |
| Zhang 2002 | ☆ | ☆ | ☆ | ☆ | - | ☆ | ☆ | ☆ | 7 |
| Hahn 2001 | ☆ | ☆ | ☆ | ☆ | - | ☆ | ☆ | ☆ | 7 |
| Grunewald 1998 | ☆ | ☆ | ☆ | ☆ | - | ☆ | ☆ | ☆ | 7 |
| Cheung 1996 | ☆ | ☆ | ☆ | ☆ | - | ☆ | ☆ | ☆ | 7 |
| Khanim 1996 | ☆ | ☆ | ☆ | ☆ | - | ☆ | ☆ | ☆ | 7 |
| Chang 1995 | ☆ | ☆ | ☆ | ☆ | - | ☆ | ☆ | ☆ | 7 |
| Jeng 1994 | ☆ | ☆ | ☆ | ☆ | - | ☆ | ☆ | ☆ | 7 |
| Abdel-Hamid 1992 | ☆ | ☆ | ☆ | ☆ | - | ☆ | ☆ | ☆ | 7 |

The “star” presents a “high-quality” choice of individual study. For high-quality study was defined as a study with ≥6 awarded stars.

Methodologic quality of cohort studies included in the meta-analysis (NOS)

| Study and year | Representa-tiveness of the exposed cohort | Selection of the unexposed cohort | Ascertain-ment of exposure | Demonstration that outcome of interest was not present at start of study | Study controls for age/gender | Study controls for additional factors | Assessment of outcome | Was follow-up long enough for outcomes to occur | Adequacy of follow up of cohorts | Total quality scores |
| --- | --- | --- | --- | --- | --- | --- | --- | --- | --- | --- |
| Tang 2008 | ☆ | - | ☆ | - | - | - | ☆ | - | ☆ | 4 |
| Ayadi 2007 | ☆ | - | ☆ | - | - | - | ☆ | - | ☆ | 4 |
| Chang 2006 | ☆ | - | ☆ | ☆ | - | - | ☆ | ☆ | ☆ | 6 |
| Nurhantari 2003 | ☆ | - | ☆ | - | - | - | ☆ | - | ☆ | 4 |
| Henry 2001 | ☆ | - | ☆ | - | - | - | ☆ | - | ☆ | 4 |
| Kuo 2001 | ☆ | ☆ | ☆ | - | - | ☆ | ☆ | ☆ | ☆ | 7 |
| D’Addario 2000 | ☆ | - | ☆ | - | - | - | ☆ | - | ☆ | 4 |
| Cheung 1998 | ☆ | - | ☆ | - | - | - | ☆ | - | ☆ | 4 |
| Sung 1998 | ☆ | - | ☆ | - | - | - | ☆ | - | ☆ | 4 |
| Bouzid 1994 | ☆ | ☆ | ☆ | - | - | ☆ | ☆ | - | ☆ | 6 |
| Miller 1994 | ☆ | ☆ | ☆ | - | - | ☆ | ☆ | - | ☆ | 6 |
| Hu 1991 | ☆ | ☆ | ☆ | - | - | ☆ | ☆ | - | ☆ | 6 |

NOS: Newcastle-Ottawa scale
