# Supplementary figures and images for: The Epstein-Barr virus latent membrane protein-1 (LMP1) 30-bp deletion and XhoI-polymorphism in nasopharyngeal carcinoma: a meta-analysis of observational studies
Source: Syst Rev. 2015 Apr 13;4:46. doi: 10.1186/s13643-015-0037-z (PMC4404015; doi:10.1186/s13643-015-0037-z)

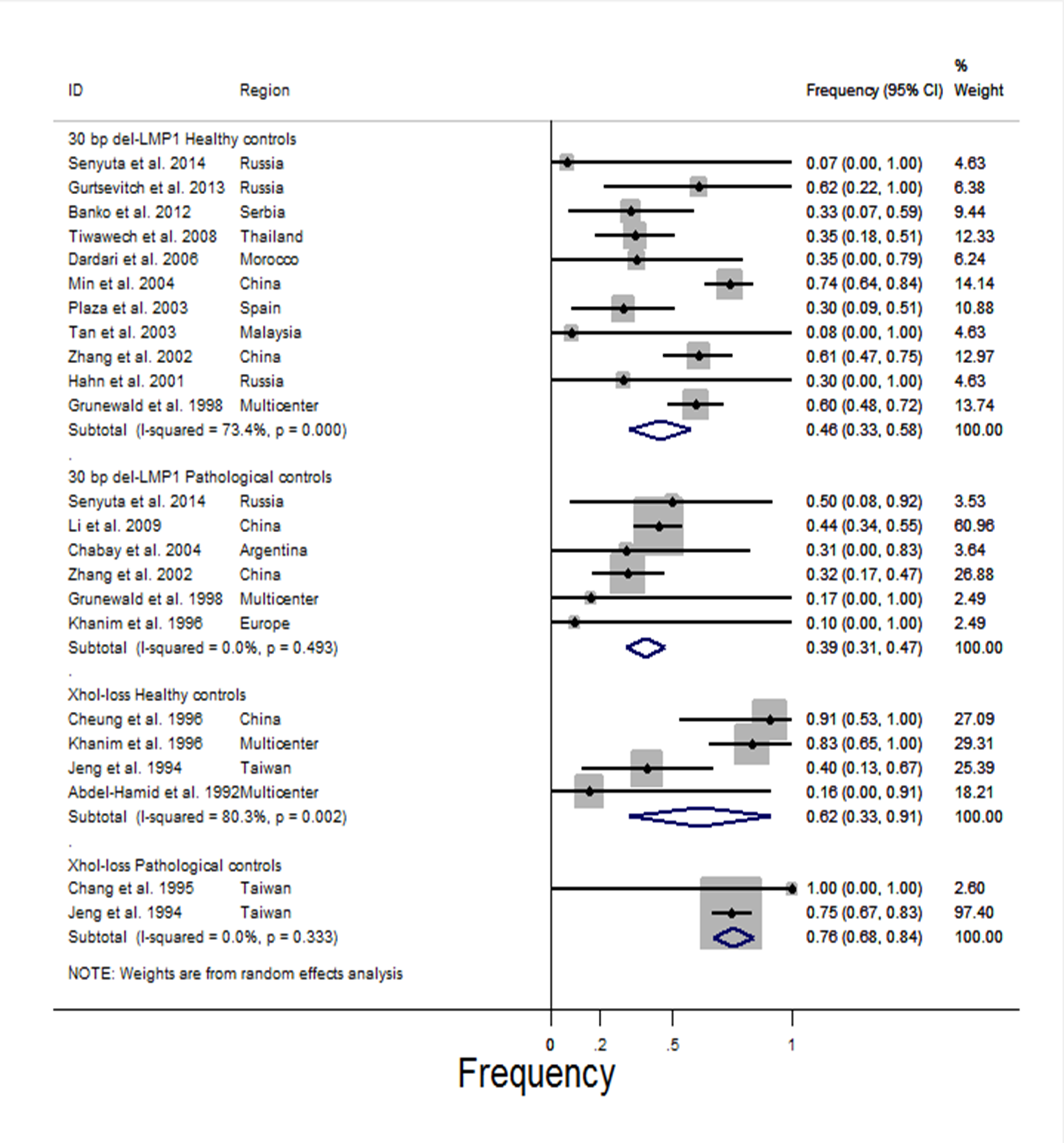

Supplement: Additional file 5: — Forest plot of the frequency of the 30-bp del-LMP1 and XhoI-loss. Forest plot of the frequency of the 30-bp del-LMP1 and XhoI-loss in the control groups. [file 13643_2015_37_MOESM5_ESM.tiff]

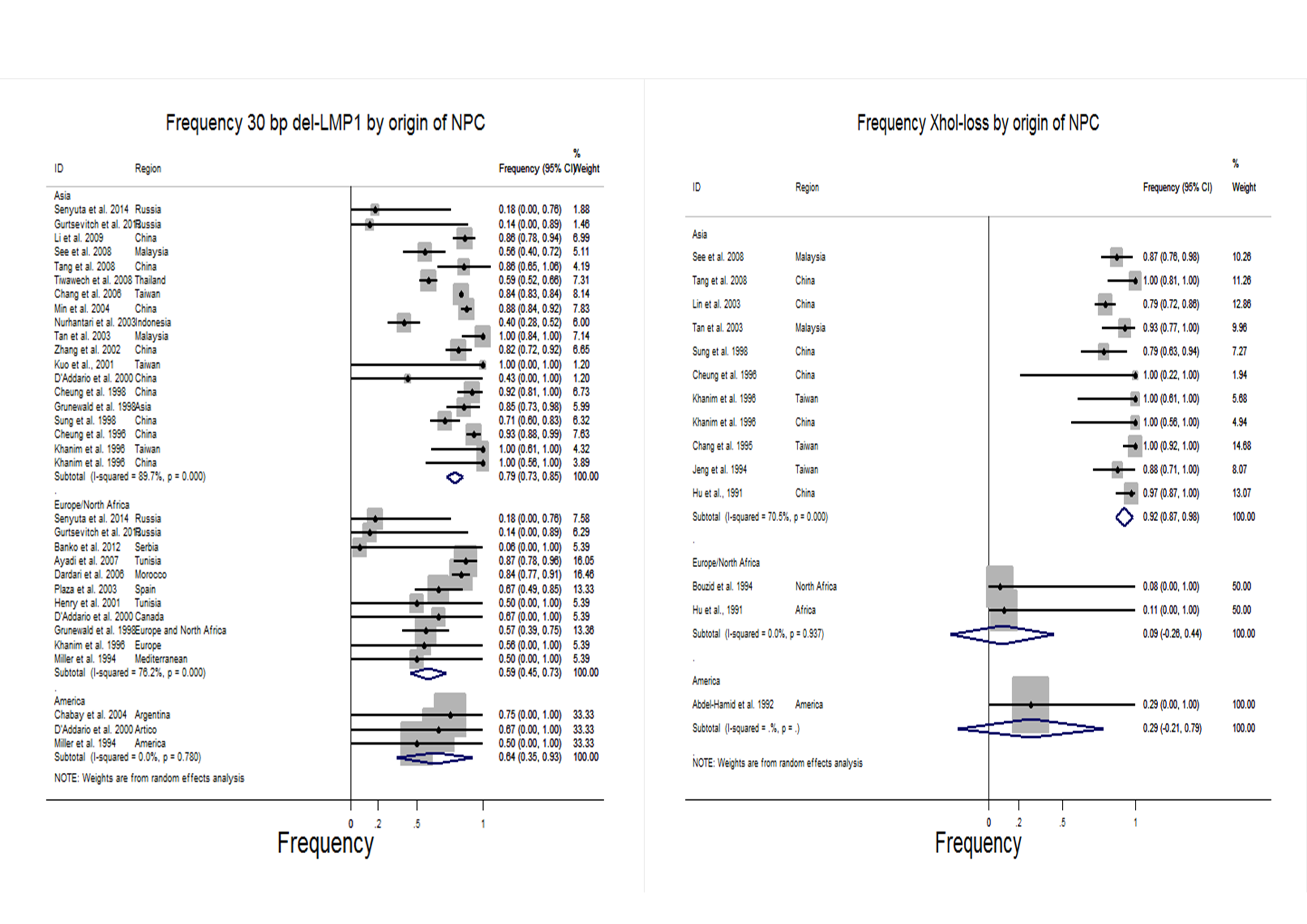

Supplement: Additional file 6: — Forest plot of the frequency of the 30-bp del-LMP1 and XhoI-loss. Forest plot of the frequency of the 30-bp del-LMP1 (left) and XhoI-loss (right) in relation to the origin from the studies. [file 13643_2015_37_MOESM6_ESM.tiff]

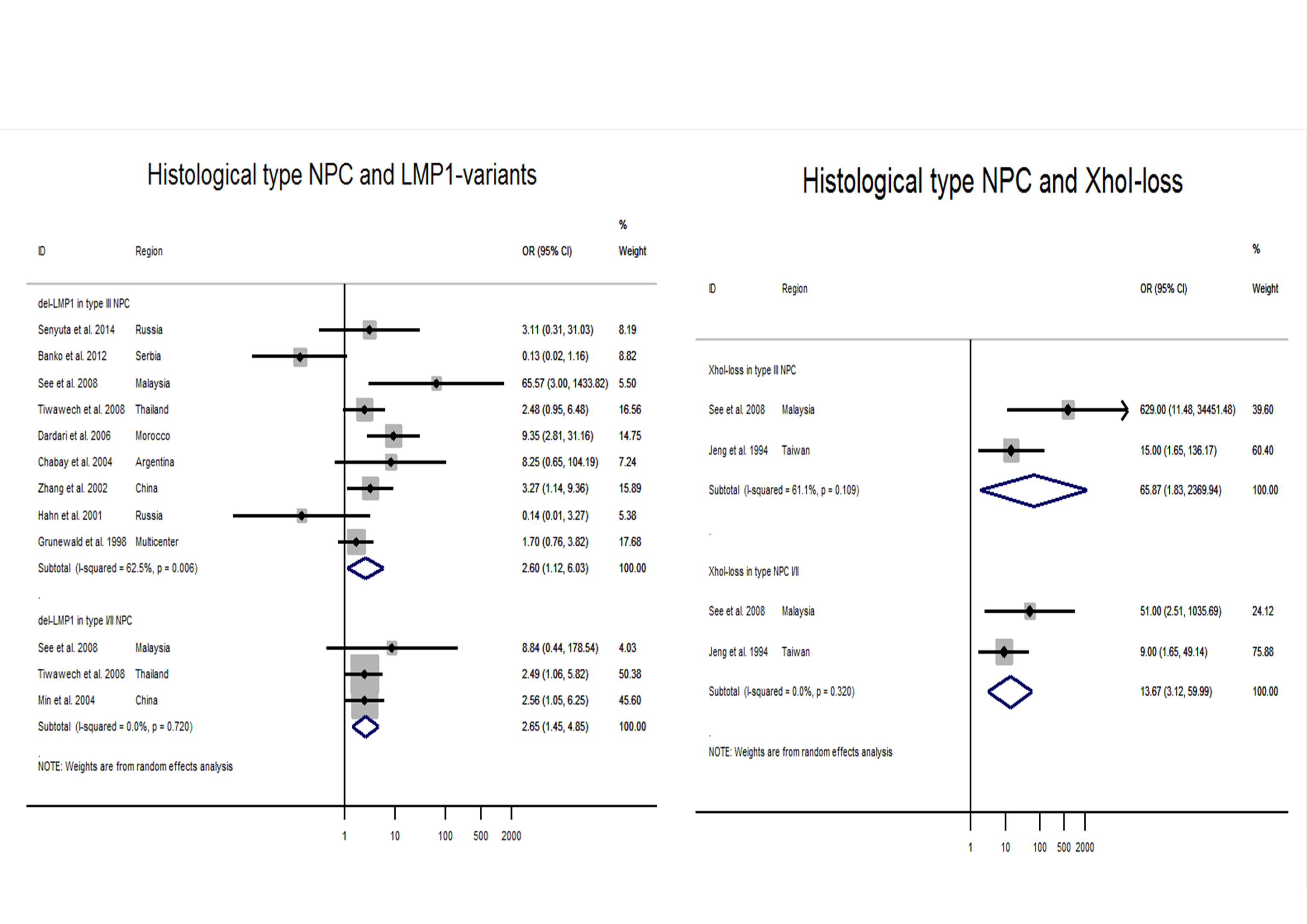

Supplement: Additional file 7: — Forest plot of the OR regarding the histological type of NPC. Forest plot of the OR regarding the histological type of NPC associated with the 30-bp del-LMP1 (left) and XhoI-loss (right). [file 13643_2015_37_MOESM7_ESM.tiff]

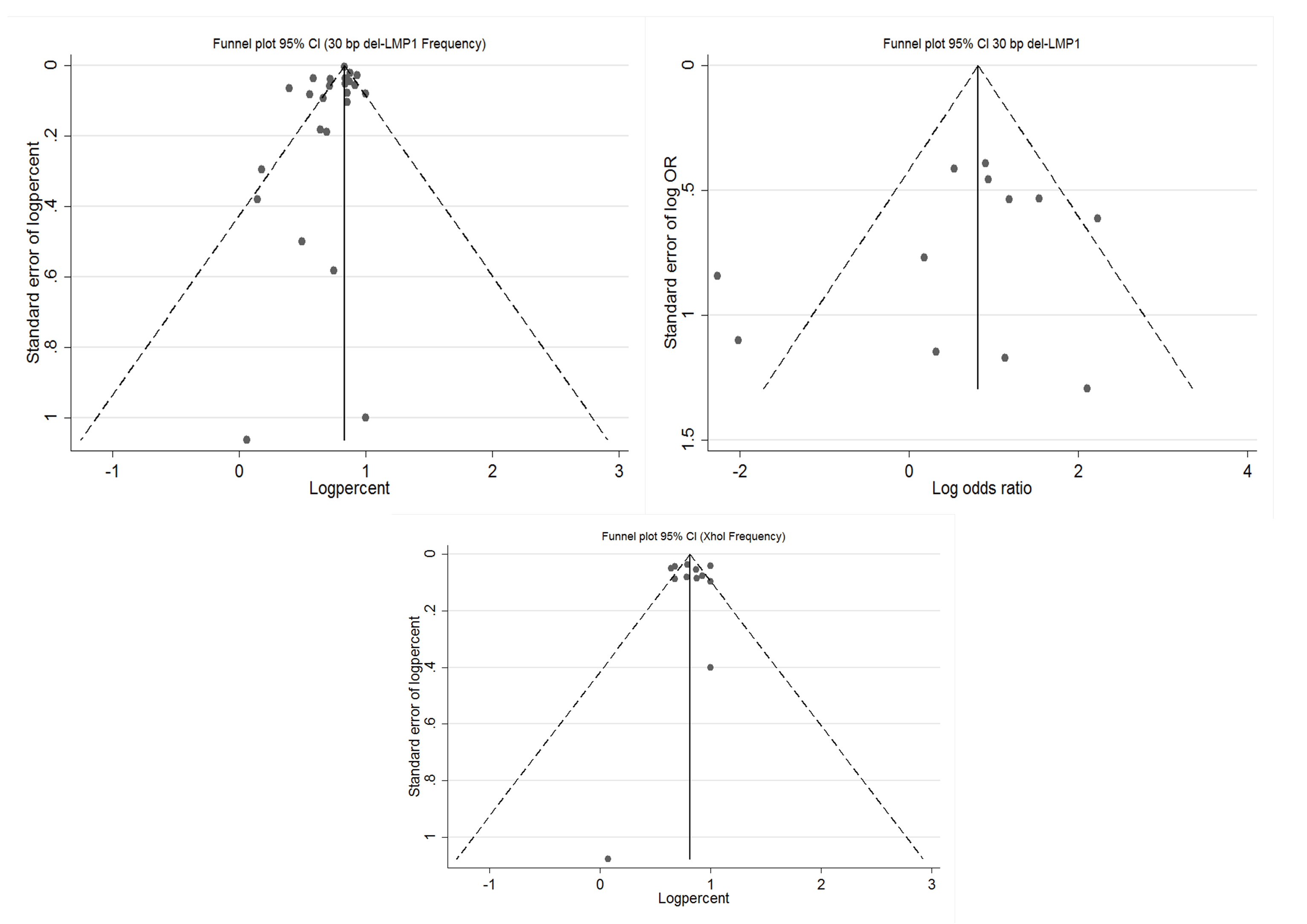

Supplement: Additional file 8: — Funnel plot for publication bias. Funnel plot for publication bias of the 30-bp del-LMP1 and Xho-loss. [file 13643_2015_37_MOESM8_ESM.tiff]
